# Data Quality Management in the Internet of Things

**DOI:** 10.3390/s21175834

**Published:** 2021-08-30

**Authors:** Lina Zhang, Dongwon Jeong, Sukhoon Lee

**Affiliations:** 1 Department of Software Convergence Engineering, Kunsan National University, Gunsan 54150, Korea; bjwllina@163.com (L.Z.); djeong@kunsan.ac.kr (D.J.); 2 School of Computer, Baoji University of Arts and Sciences, Baoji 721000, China

**Keywords:** data quality, data quality management, data quality methodology, data quality standard, IoT data

## Abstract

Nowadays, IoT is being used in more and more application areas and the importance of IoT data quality is widely recognized by practitioners and researchers. The requirements for data and its quality vary from application to application or organization in different contexts. Many methodologies and frameworks include techniques for defining, assessing, and improving data quality. However, due to the diversity of requirements, it can be a challenge to choose the appropriate technique for the IoT system. This paper surveys data quality frameworks and methodologies for IoT data, and related international standards, comparing them in terms of data types, data quality definitions, dimensions and metrics, and the choice of assessment dimensions. The survey is intended to help narrow down the possible choices of IoT data quality management technique.

## 1. Introduction

The concept of the Internet of Things (IoT) was first introduced by Ashton [1] to describe the ability of sensors to connect to the Internet and provide new services. Ma [2] also defined the IoT as a network that connects an ordinary physical object with an identifiable address to provide intelligent services. After decades of development, multiple objects around us can be connected through networks and these connected objects can share data that have and will affect various fields.

The report shows that there were 620 known IoT platforms by the end of 2019, more than twice as many as in 2015 [3]. Meanwhile, the growing number of IoT platforms are generating exponentially more data and speed. By 2025, the total amount of data from connected devices worldwide is expected to reach 79.4 Zettabytes (ZBs) [4].

As the scale of the IoT increases, the probability of system and network failure of the IoT increases significantly. These errors can lead to poor sensor data quality (DQ) and can lead to incorrect decision outcomes [5]. DQ has been extensively studied and is becoming one of the more mature research fields. A comprehensive survey of data evaluation techniques and improvement techniques was conducted by Batini et al. [6]. Because the IoT is still young, although many studies have proposed DQ management methodologies, frameworks, ISO standards and tools, the importance of IoT DQ has been largely overlooked. Karkouch et al. [7] provided an overview of the state of the art in IoT DQ management to find solutions to enhance DQ. Sathe et al. [8] surveyed model-based sensor data collection and management techniques. The survey by Qin et al. [9] investigated some key research areas in the IoT from a data-centric perspective and did not specifically focus on DQ assessment techniques. Liu et al. [10] reviewed and analyzed 45 studies of DQ in the IoT from 1999 to 2018, focused on the dimensions of DQ and approaches to measure them. The IoT is the source of a lot of Big Data (device data), and the analysis of a lot of device data depends on Big Data. Taleb et al. [11] investigate, classify, and discuss the latest work on Big Data quality management and perform quality assessment practices for different Big Data phases.

IoT systems inherently collect data from heterogeneous sources, and various types of sensors can present different data precision, data ranges, units, device specifications, etc. [12], and with inherent uncertainties. DQ is such a very subjective concept that different applications and different data types have various needs for DQ requirements. However, existing surveys related to DQ do not have recommendations on the choice of assessment methods for the diversity of IoT data types. To address this requirement, we surveyed the current state of IoT DQ management. First, we summarize the definitions of data types and DQ, then identify DQ issues for IoT platforms. Further, an overview of DQ management methodologies and frameworks that can be customized for IoT data, international standards and IoT DQ management techniques is presented. Finally, we list the commonly used dimensions and compare them. We aim to provide an overview of the current DQ of IoT platforms to find suitable methods for assessing IoT data.

The remainder of this article is organized as follows: Section 2 discusses the data types, the concept of DQ and IoT DQ issues. In Section 3, we review some techniques for DQ management. Section 4 defines the dimensions, gives the calculation of the metrics for the corresponding dimensions, compares the studies designed in the survey and finally discuss how to select proper dimensions. Finally, Section 5 provides conclusions and future work.

## 2. Data Quality in IoT

It is evident from previous research [6] that there is usually an important phase in the initial stage of the DQ management technique that is used to define the data and DQ. This includes the context and type of data. In addition, analyzing the factors in the system that potentially affect DQ has an impact on DQ assessment. This chapter summarizes the classification of general data and IoT data, describes the definition of DQ, and provides a discussion of the potential factors that affect IoT DQ.

### 2.1. Definition of Types of Data

Data are “abstract representations of selected characteristics of real-world objects, events, and concepts, expressed and understood through explicitly definable conventions related to their meaning, collection, and storage” [13]. The term information is used in some studies and is interchangeable with data, without clear distinction. In [14], the authors use data to denote structured data in databases, and other broader types of data are described by information except linked open data, and Big Data. In some studies [15,16], the generic term information is used to indicate that the study may involve any type of data, without specifying a specific type of data. Data can be categorized into different types depending on how they are used in different areas. Researchers proposed several classifications of data, as shown in Table 1. In the existing studies, the most widely used data classification is that based on data structure.

The International Organization for Standardization (ISO)000-2 [17] defines data as “an interpretive representation of information in a appropriate format”. ISO 8000-100 [18] lists special data including but not limited to: master data, transaction data, and measurement data. Master data are data that an organization hold to characterize entities that are both separate and essential to that organization and are cited for the purpose of executing its transactions, consist of reference data and feature data. Transaction data are data that represents business transactions. Measurement data are the data to record the measurement results.

IoT data collects smart things over networks, with special characteristics such as distribution, volume, velocity, variety, veracity, value, spatio-temporality and dynamicity. Different characteristics and different sources of IoT data may have different data management methods. Before carrying out DQ management or assessment, it is very important to determine the type of data. Fathy et al. summarized the three classifications of IoT data, and explained each category, including numerical (referring to data with numerical values) vs. symbolic (data that have string/text values), discrete (data with finite values or finite sets of values) vs. continuous (data with unlimited sets of values), and static(data that do not change) vs. streaming(data that changes over time) [19,20]. Klein et al. [21] present findings that show streaming data includes the numerical, discretized and digitized data. Cooper and James classified IoT data into Radio Frequency Identification (RFID) address/unique identifiers, descriptive data, positional and environmental data, sensor data, historical data, the physics model, and command data based on domain [22].

**Figure 1:**
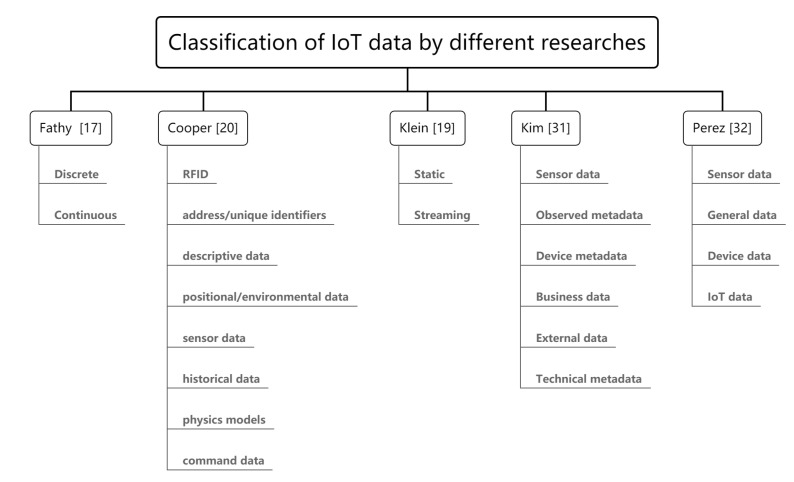
Classification of IoT data by different research.

From the perspective of data semantics, the data in the IoT can be the underlying raw data and the high-level generalized data. Different data formats introduce the basis of data polymorphism and data heterogeneity [32]. Kim et al. [33] divide the data generated and used in the IoT into six categories, including sensor data (sensor-generated data); observed metadata (describe sensor data behavior); device metadata (describe the characteristics of the device or sensor); business data (for business purposes); external data (provide additional information for product capabilities, such as weather) and technical metadata (data standards and physical data storage structures). Perez-Castillo et al. consider the dependence on various data sources and classify data involved in the IoT into four categories [34]: sensor data, which is generated by sensors and digitized into machine-readable data (For example, the reading of temperature sensors); device data: metadata of sensor observations and IoT devices (for example, the timestamp of the observation and the device manufacturer); general data: IoT-device-generated or device-related data (for example, sensor observations stored in a database); IoT data: in an IoT system, all data other than the raw data generated by sensors are collectively referred to as IoT data, which is a collection of general data and device data. Many studies have been published on sensor DQ [12,20,35,36,37,38,39,40,41,42] and streaming data DQ management [20,21,36,43]. We summarize various classification methods for IoT data mentioned in the literature, as shown in Figure 1.

### 2.2. Definition of Data Quality

DQ has been defined differently in various fields and time periods. The understanding of the concept of DQ mainly includes the following two perspectives: first, it focuses on measuring DQ from a practical perspective, i.e., it is judged from a user perspective, emphasizing user satisfaction, and also from data producers and managers; second, it focuses on evaluation from a system-oriented perspective, considering DQ as a comprehensive concept and a multidimensional concept. It is necessary to measure its basic quality elements from multiple perspectives, such as accuracy, timeliness, completeness, and consistency.

One of the first people to define a basis DQ was Wang et al. [44], who wrote: “data that are fit for use by data consumers”. Juran et al. [45] provided a new definition of DQ: “data are of high quality if they are fit for their intended uses in operations, decision making, and planning”. The ISO 9000 standard [46] defines quality as “the degree to which a set of inherent characteristics fulfills a need or expectation that is stated, generally implied, or obligatory”.

The ISO 8000-2 [17] present DQ as the degree to which the inherent characteristics of data meet the demands. The ISO 8000-8 classifies information and DQ into three categories: syntactic quality, semantic quality and pragmatic quality. Syntactic quality refers to the extent to which the data conform to the specified syntax, such as consistency with metadata. Semantic quality describes how well the data correspond to the content it stands for. Pragmatic quality refers to the extent to which the data are appropriate and valuable for a specific objective [47]. As defined by the ISO 8000, DQ includes the following principles:

- The data are fit for purpose;
- Being in the right place at the right time, with the right data;
- Meet the data requirements agreed by the customer;
- Preventing duplication and eliminating waste through enhancement phases and preventing the recurrence of data defects.

The definition of IoT DQ is basically aligned with the definition of DQ mentioned above. A further definition is given by Karkouch [7], who describes whether the data collected (from IoT devices) is appropriate for IoT users to provide ubiquitous services. IoT devices typically monitor a variable of interest in the physical world, such as temperature, sleep habits, and so on.

DQ research and practices can be categorized into top–down and bottom–up approaches [48]. The top–down approach usually proposes a DQ framework with DQ dimensions, and then by integrating with specific requirements in the application, more detailed DQ dimensions are constructed, while the bottom–up approach starts by refining a series of DQ dimensions from specific requirements, and through the demonstration of practical applications, the DQ framework is finally generalized.

### 2.3. Issues of Data Quality

Data suffering from quality issues are not representative of the true situation and may negatively impact the decision making and operational levels of any business or organization. The challenges facing the IoT data directly inherit and even amplify the characteristics of the Internet because of large-scale deployments of IoT devices, information flows, and indirect user involvement [49].

Lee et al. concluded ten root causes of DQ problems: multiple data sources, subjective judgments during data generation, insufficient computational resources, the balance of security and accessibility, cross-disciplinary encoding of data, complex data representation, data volume, input rules that are overly restrictive or ignored, evolving data demands, and distributed heterogeneous systems, respectively [50]. These ten root causes are equally applicable in IoT systems. Jeffery et al. [35] summarized two types of DQ problems “missed readings” and “unreliable readings” generated by the IoT devices. For example, the sensor average delivery is only 42% in an IoT experiment, which will lead to dropped data. IoT data may come from multiple different objects and have different formats, which will lead to inconsistencies in multi-source data [51]. Additionally, problems such as data duplication [52], data leakage, and time calibration of multiple data sources were reported in the studies.

**Figure 2:**
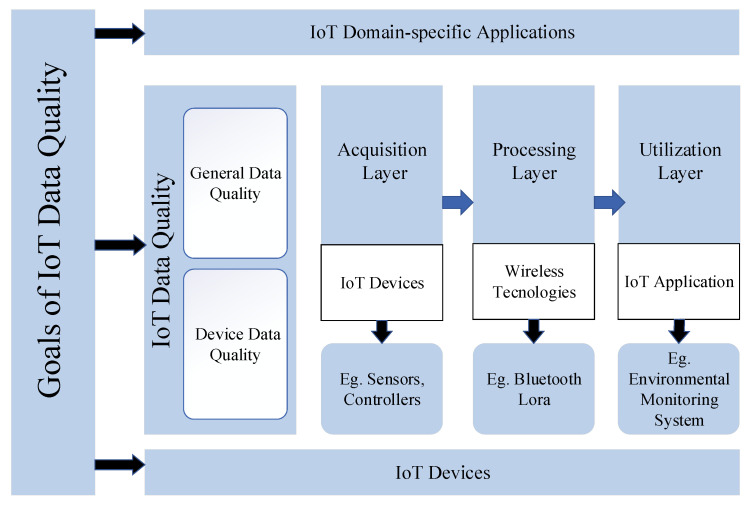
IoT DQ conceptual framework [57].

To better examine and appreciate the DQ problems and challenges in the IoT, we describe the features and problems of the IoT via a three-layer structure [53]. A typical three-layer IoT system consists of the perception layer, the network layer, and the application layer. In the perception layer, which is also known as the device layer [54], the physical objects and IoT devices, such as DHT11, which includes temperature and humidity sensors, measure and collect the observed temperature and humidity results. Next, the network layer is used to send the observation results via wireless technologies, such as LoRa [55] and Bluetooth. Then, the application layer receives observation results from the previous layer, where data processing, analysis, and storage are all carried out, and provide users with ubiquitous services. Perez-Castill et al. propose a three-layer conceptual framework for IoT DQ, as shown in Figure 2, with each layer focusing on both device DQ and general DQ [56,57].

Many researchers have found that DQ problems may occur in different layers of the IoT structure and affect the DQ of IoT platform, which are: the deployment scale, resource constraints, network, sensors, environment, vandalism, fail-dirty, privacy preservation processing, security vulnerability, and data stream processing [7,8,20,41,58,59,60,61,62,63,64].

Teh et al. identified eight types of sensor data errors: anomalies, missing values, deviations, drift, noise, constant value, uncertainty, and stuck-at-zero [5]. The most common error is outliers, values that lie above the thresholds or significantly deviate from the normal behavior provided by the model. The second most common error in sensor data is missing data, also known as incomplete data in relational databases. Li and Parker [65] believed that missing data are due to a variety of factors, such as an unstable wireless connection caused by network congestion, the power failure of sensor devices caused by limited battery life, and environmental interference such as artificial blockage, walls, weather conditions and vandalism.

In each layer of the data transmitting process, there may be different DQ issues due to various impacts. As shown in Table 2, we summarized causes and the error types that may result from each layer [7,66], which should be detected and corrected to improve IoT DQ. While some issues affect only one layer, many cross multiple layers.

## 3. Data Quality Management Techniques Review

This chapter discusses the DQ management techniques, including methodologies and frameworks. Several DQ management methodologies have been applied and validated in industry and research and can be directly applied to IoT platforms. The study [6] considers DQ methodologies as a set of criteria and technologies that define a rational process for assessing and improving DQ, starting with input information that describes the context of a given application.

This review builds most initially on other surveys [7,67,68,69] and reviews [5,10,70]. Two of the most important surveys, including (1) Batini et al. [6], summarize 13 methodologies for DQ assessment and improvement up to 2009, and (2) Cichy et al. [24] summarize 12 methodologies/frameworks applicable to information systems or business data published up to 2020, and 13 special-purpose DQ frameworks (including two ISO standards). After counting and removing duplicate studies, we obtained a total of 32 DQ management methodologies, standards and frameworks. In the second step, frameworks that can only be applied to specific domains and not to the IoT domain were screened out. In the second step, a total of 18 methodologies/frameworks that could only be applied to specific domains rather than the IoT domain were screened out, leaving 12 general IoT DQ management methodologies/frameworks, and 2 ISO standards. In the third step, the keywords “IoT data quality” and “methodology or framework” were searched in Google Scholar, and nine frameworks and methodologies for IoT DQ assessment before 2021 were obtained after reading the abstracts. In the last step, after reading and filtering the references of these studies, three additional ISO standards related to DQ were obtained, adding the two previous ones, making a total of five. Finally, a total of 21 studies and 5 ISO standards were screened out that met the requirements. Next, we compare the main stages and evaluation techniques, and the results of the comparison will guide developers or users of the IoT platform to choose appropriate data management or evaluation methods.

### 3.1. Methodology and Frameworks Appropriate for IoT Data

Batini et al. defined a DQ methodology [6] as “a set of guidelines and techniques that define a rational process for assessing and improving DQ, starting from describing the input information for a given application context, defines a rational process to assess and improve the quality of data”. A framework is considered as a theory-building and practice-oriented tool [71], providing a structure for using QA theory and methods [72,73]. The terms DQ methodology and DQ framework are often used interchangeably in related research. In this chapter, we review the general DQ management methodologies and frameworks, comparing them in terms of research objectives, management phases, applicable data types, the number of DQ dimensions, and whether they can be extended, respectively. Most of the research in DQ methodology has focused on structured and semi-structured data, while only a few of them also involve semi-structured data. Methodology and framework, in many studies, refer to the same thing.

Early on, Wang [74] proposed a general methodology “Total Data Quality Management (TDQM)”, which is one of the most famous complete and general methodologies. The TDQM treats data as information products and presents a comprehensive set of associated dimensions and enhancements, which can be applied to different contexts. However, the structure of the processable data is not specified. The goal of TDQM is to continuously enhance the quality of information products through a cycle of defining, measuring, analyzing and enhancing data and the process of managing them, without appropriate steps specified in the assessment process.

English [15] described a methodology of “Total Information Quality Management (TIQM)” applied to data warehouse projects. Later, due to its detailed design and universality, it became a generic information quality management methodology that can be customized for many backgrounds and different data types, including structured data, unstructured data, and semi-structured data, the latter two of which are not mentioned in the study but can be inferred. The TIQM cycle includes evaluation, improvement, and improvement management and monitoring. Compared with other methodology, TIQM is original and more comprehensive in terms of cost–benefit analysis and the management perspective [14]. However, during the evaluation phase, TIQM manages a fixed set of DQ dimensions, with a number of DQ dimensions of 13 and their solution strictly follows these dimensions. TIQM is one of the few methodologies that considers the cost dimension and provides detailed classifications for costs.

Lee et al. [16] presented “A Methodology for Information Quality Assessment (AIMQ)”, which is the first quality management method that focuses on benchmarking and will provide objective and domain-independent generic quality assessment techniques. The methodology designs a PSP/IQ model that provides a standard list of quality dimensions and attributes that can be used to categorize quality dimensions according to importance from a user and an administrator perspective. The AIMQ cycle includes the measurement, analysis, and interpretation of an assessment, and lacks guidance on activities to improve DQ. AIMQ uses questionnaires applicable to structured data for qualitative assessments but can be applied to other data types, including unstructured data and semi-structured data. Similar to TIQM, during the measurement phase, AIMQ manages a fixed group of DQ dimensions (metrics), with a number of dimensions of 15, and their solution strictly follows these dimensions.

Monica et al. [75] present a cooperative framework “DaQuinCIS” for DQ by applying TDQM, which is one of the rare methodologies that focuses on semi-structured data. This approach proposes a model, called data and data quality (D2Q). The model associates DQ values with XML documents, and can be used to verify the accuracy, currency, completeness, and consistency of the data. Another contribution of DaQuinCIS is the degree of flexibility that each organization has to export the quality of its data because of the semi-structured model.

Batini et al. [27] proposed a “Comprehensive Data Quality methodology (CDQ)” that extends the steps and techniques originally developed for all types of organizational data. CDQ integrates the phases, techniques and tools from other methodologies and overcomes some of the limitations in those methodologies. The CDQ cycle includes state reconstruction, assessment, and improvement. All data types, both structured and semi-structured, should be investigated in the state reconstruction step. CDQ manages four DQ dimensions and considers the cost of alternative improvement activities to compare and evaluate the minimum-cost improvement processes.

Cappiello [76] described a “Hybrid Information Quality Management (HIQM) methodology”, which supported error detection and correction management at runtime and improved the traditional DQ management cycle by adding the user perspective. For example, HIQM defines DQ by considering the needs of not only companies and suppliers, but also user end consumers to determine DQ requirements. The HIQM cycle includes definition, quality measurement, analysis and monitoring, and improvement. However, in the measurement stage, only the need for measurement algorithms for each DQ dimension is expressed, without defining specific metrics. In particular, TIQM designed a warning interface that represents an efficient way to analyze and manage problems and warnings that appear, and considers whether to recommend a recovery operation by analyzing the details of the warning message.

Caballero [77] proposed “A Methodology Based on ISO/IEC 15939 to Draw up Data Quality Measurement Process (MMPRO)”, which is based on the ISO/IEC 15939 standard [78] for software quality assessment and can also be used for DQ assessment. The MMPRO cycle includes the DQ Measurement Commitment, Plan the DQ Measurement Process, Perform the DQ Measurement Process and Evaluate the DQ Measurement Process. Although the approach does not categorize DQ measures or provide a set of behaviors for improving data quality, its structure helps to incorporate DQ issues into the software.

Maria et al. [79] described “A Data Quality Practical Approach (DQPA)”, which described a DQ framework in a heterogeneous multi-database environment and applied it with a use case. The DQPA cycle consists of seven phases, the including identification of DQ issues, identification of relevant data that has a direct impact on the business, evaluation, the determination of the business impact through DQ comparison, cleansing of data, monitoring the DQ, and carrying out the assessment stage regularly. In DQPA, the authors propose the Measurement Model based on [80,81,82], which extends the DQ assessment metrics into metrics for evaluating primary data sources and metrics for evaluating derived data. The model can be used at different levels of granularity for databases, relationships, tuples, and attributes.

Batini et al. [28] presented a “Heterogenous Data Quality Methodology (HDQM)”, which can be used to evaluate and improve the DQ, and has been verified by using cases. The HDQM cycle includes state reconstruction, assessment, and improvement. The HDQM recommends considering all types of data in the state reconstruction phase by using a model that describes the information according to the level of abstraction. In the assessment phase, HDQM defines a method that can be easily generalized to any dimension. Furthermore, the DQ dimensions of the HDQM measurement and improvement phase can be applied to different data types. A major contribution of HDQM is based on the techniques of the cost–benefit analysis in TIQM, COLDQ and CDQ, presenting a more qualitative approach to guide the selection of appropriate improvement techniques.

Laura et al. [13] described a “Data Quality Measurement Framework (DQAF)”, which provides a comprehensive set of objective DQ metrics for DQ assessment organizations to choose from, comprising 48 universal measurement types based on completeness, timeliness, validity, consistency, and integrity. In DQAF, the authors introduce a concept of “measurement type” that is a generic form suitable for a particular metric, and develop some strategies to describe six aspects of each measure type, including definition, business concerns, measurement methodology, programming, support processes and a skills and measurement logical model. The DQAF cycle includes define, measure, analyze, improve, and control. Specifically, the authors focus on comparing the results of the DQ assessment with assumptions or expectations, and continuously monitoring the data to ensure that it continues to meet the requirements.

Carretero et al. [83] developed an “Alarcos Data Improvement Model (MADM Framework)” that can be applied in many fields, which can provide a Process Reference Model and evaluation and improvement methods. Finally, it was verified with an applied hospital case. The MADM Framework cycle consists of a two-stage Process Reference Model based on the ISO 8000-61 standard and an Assessment and Improvement Model based on ISO/IEC 33000. The MAMD Process Reference Model consists of 21 processes that can be used in the areas of data management, data governance, and DQ management quality. The assessment model is a methodology that consists of five steps and a maturity model.

Reza et al. [84] introduced an “observe–orient–decide–act (OODA)” framework to identify and improve DQ through the cyclic application of the OODA method, which is adaptive and can be used across industries, organizational types, and organizational scales. The OODA framework cycle includes observe, orient, decide and act. Only the need for a metric algorithm for each DQ dimension is indicated, and the OODA DQ approach refers to the use of existing DQ metrics and tools for metrics. Although the OODA DQ methodology does not involve any formal process for analysis and improvement processes, DQ issues are identified through tools such as routine reports and dashboards during the observe phase. In addition, notices alerting for possible DQ issues and feedback from external agencies are also recommended [24].

There are many more comparative perspectives on these 12 general DQ management methodologies/frameworks, such as flexibility in the choice of dimensions [24], the use of subjective or objective measurements in the assessment phase, specific steps in the assessment/improvement phase, cost considerations, data-driven or process-driven, etc. There is not much research on IoT DQ assessment yet, and a beginner may have some difficulties on aspects such as how to make decisions, so start with the question, what are the general requirements of data users? If the user needs to manage IoT data in a holistic way that supports the definition, assessment and improvement process without resorting to some tool or software, the generic DQ management methodology/framework mentioned in this section can be chosen.

### 3.2. ISO Standards Related to Data Quality

Another important area of DQ in industry and academia is the research and standardization of DQ standards. By developing uniform DQ standards, DQ can be managed more efficiently across countries, organizations, and departments, thereby facilitating data storage, delivery, and sharing, and reducing errors in judgment and decision making due to data incompatibility, data redundancy, and data deficiencies. Since IoT systems are distributed in nature, the use of international standards can have a positive effect on improving the performance of business processes by aligning various organizations with the same foundation, addressing interoperability issues, and finally working in a seamless manner.

The ISOas made a great deal of effort in this regard and has developed several standards to regulate international data quality. The ISO 8000 DQ standard has been developed [85] to address the increasingly important issue of DQ and data management. ISO 8000 covers the quality characteristics of data throughout the product life cycle, from conceptual design to disposal. ISO 8000 describes a framework for improving the DQ of a particular data, which can be used independently or in cooperation with a quality management system.

**Figure 3:**
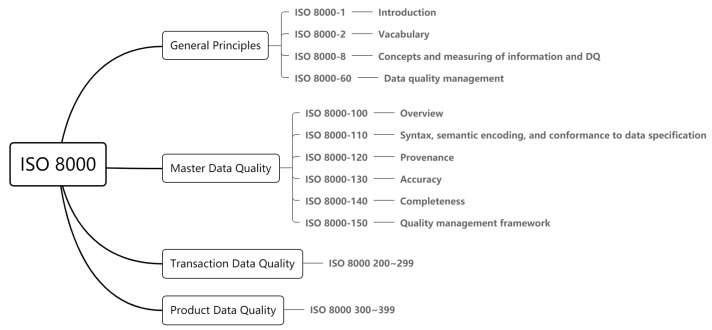
Components of the ISO 8000.

The ISO 8000-6x family of standards provides a value-driven approach to DQ management. Several of the IoT data assessment frameworks reviewed in the next section are based on this standard. This series of standards provides a set of guidelines for the overall management of DQ that can be customized for different domains. It describes a DQ management structure derived from ISO 9001’s Plan-Do-Check-Act (PDCA), a life cycle that includes DQ planning, DQ control, DQ assurance, and DQ improvement. However, it is not primarily intended as a methodology for DQ management, but merely to serve as a process reference model. Figure 3 depicts the components of the ISO 8000 DQ standard.

Before ISO 8000 DQ standards were published, a more mature management system of product quality standards existed—ISO 9000 [86]. Initially published by the ISO in 1987 and refined several times, the ISO 9000 family of standards was designed to help organizations ensure that they meet the needs of their customers and other stakeholders while meeting the legal and regulatory requirements associated with their products. It is a general requirement and guide for quality management that helps organizations to effectively implement and operate a quality management system. While ISO 9000 is concerned with product quality, ISO 8000 is focused on DQ. ISO 8000 is designed to improve data-based quality management systems, a standard that addresses the gap between ISO 9000 standards and data products [87].

In addition, international standards related to DQ include ISO/IEC 25012 Software Product Quality Requirements and Assessment Data Quality Model [88], ISO/IEC 25024 Quality Requirements and Evaluation of Systems and Software [89]—Measurement of Data Quality, etc. ISO/IEC 25012 standard proposes a DQ model called Software Product Quality Requirements and Evaluation (SQuaRE) that can be used to manage any type of data. It emphasizes the view of DQ as a part of the information system and defines quality features for the subject data. In the following, we compare the following 5 ISO standards that are often used in DQ management studies, as shown in Table 3.

The benefits of customizing and using international standards in the IoT context are: (1) the number of issues and system failures in the IoT environment will be reduced and all stakeholders will be aligned. (2) It is easier to apply DQ solutions on a global scale due to reduced heterogeneity. (3) DQ research in the IoT can be aligned with international standards to provide standardized solutions. (4) It enables better communication between partners.

### 3.3. IoT Data Quality Methodologies and Frameworks

As described in the previous two sections, although many studies proposed DQ techniques, the DQ of the IoT has not been extensively studied because of the youth of IoT. Additionally, most research is concerned with enhancing the quality of sensor data rather than the IoT data that includes various types of data. Based on the type of data of interest, the studies dealing with the issue of DQ management for IoT data are divided into two types, one type are about SCP and IoT environment, and the other are about sensor data streams.

Klein et al. [20] propose a meta model for managing streaming data and static DQ that can propagate DQ from the perception layer of IoT to the application layer without significant data overhead. In the paper, the authors extend the traditional Relational Database Management System (RDBMS)eta model to store DQ information persistently in a relational database. In this study, only two dimensions of DQ, accuracy and completeness, were considered. In 2009, the authors proposed another approach for data streaming quality management [21]. For the comprehensive evaluation of sensor measurements, DQ in the context of streaming data was defined and five DQ dimensions were proposed: accuracy, confidence, completeness, data volume, and timeliness.

Togt et al. [90] presented a generic framework to describe a system approach that can be evaluated to assess RFID system DQ and performance in a specific healthcare environment. The framework is composed of nine stages, including execution schedule, RFID and medical device interference testing, accuracy and integrity testing, simulated field and real field setups. This framework focuses on evaluating performance, and DQ is only tested for accuracy and completeness, and more DQ dimensions need to be added in the testing phase.

D’Aniello et al. [91] proposed a quality-aware sensor data management framework as a middleware, designing a virtual sensor that allows multiple users to determine their own quality claims through the same virtual sensor. This framework developed semantic modeling for quality-aware sensor management, using ontologies to represent sensors and data quality, and fuzzy logic to evaluate the quality of the received data. The core of the virtual quality-aware sensor is a semantic layer and a process for assessing the quality of each sensor reading.

Geisler et al. [43] applied TDQM methodology to design a DQ quality management framework for IoT data streams based on ontology, and evaluated it in the fields of the transportation system and health monitoring, proving the flexibility and effectiveness of the framework. The most important contribution of this DQ framework is the development of an ontology for managing DQ-related metadata, which includes data sources, DQ factors and DQ metrics. The approach classifies DQ metrics into three types: content-based metrics, query-based metrics, and application-based metrics, which can be implemented through various methods such as semantic rules. This framework has also been extended to the Global Sensor Network system.

To solve the shortcomings of ISO/TS 8000-61 and generic DQ management methodology, Perez et al. [34] proposed “an ISO 8000-61 Based Data Quality Management Methodology for Sensor Data” (Daqua-Mass) for SCP-based environments. It is built according to the PDCA cycle of continuous improvement and proposes the DAQUA-model, which is the core of the PDCA cycle. The model is derived from ISO/IEC 25012 and consists of several DQ features suitable for data problems to identify and represent the DQ requirements needed in the environment. The methodology is composed of seven steps, which are divided into several activities, each of which identifies the input and output products, and the various roles involved in the quality management of sensor data are identified. Aligned with ISO/IEC 25012, the 15 DQ characteristics and common sensor data errors are summarized, and each sensor data error is mapped to the primary and secondary effects on the DQ characteristics.

Perez-Castillo et al. [57] studied the ISO/IEC 25000 and ISO 8000 series of international standards and proposed a method for measuring, managing, and improving DQ in an IoT environment (DQIoT). This framework proposes an IoT DQ conceptual framework applicable to different types of IoT systems, and states that both device DQ and general DQ should be considered at the perception, network, and application layers of the IoT. The DQIoT framework lists the 23 best practices of this framework to provide ideas for researchers and managers of DQ in the IoT. However, the ISO 8000-61 based approaches do not take into account existing DQ management standards, which have not been customized for the IoT environment, and are subject to further validation.

Kim et al. [33] propose a process-centric data DQ management (DQM) framework for the IoT-based ISO 8000. The framework extends the Data Quality Management (DQM) Process Reference Model (PRM) and is customized to fully accommodate the specific requirements of IoT data management. Seven procedures are presented that are required for SCP operation and can be used for sensor data management in the product cloud. The proposed IoT DQM-PRM is useful in improving the quality of IoT data, and in processing real-time streaming sensor data and other structured and semi-structured data.

Alrae et al. [56] propose a framework which systematically managed the overall information quality (IQ) of the IoT system, and verified the proposed framework through comparative studies. The framework views information like a product of an IoT system; the DQ dimensions are the constituent parts of that product, and IoT technology elements are viewed as technical requirements of that product, and the framework uses House of Quality (HoQ) techniques to correlate DQ dimensions with IoT technology elements. The framework’s IQ Management Processes include assessment, awareness, and action. It is only applicable to small IoT systems; the validation phase is time consuming and has only been tested against other cases and needs further improvement.

## 4. Data Quality Dimensions and Metrics in IoT

The current prevailing view is that DQ elements are greatly influenced by factors such as the industry domain, data type and application purpose, and there is no universal DQ indicator system for all subject areas and resource types, but it is perfectly feasible to establish a set of publicly recognized quality dimensions and metrics systems and specify their collection methods for a specific data type in a specific industry background. This chapter introduces the commonly used DQ dimensions in the IoT domain and compares the use of these dimensions in practical research.

### 4.1. Definition of Dimensions and Metrics

A DQ dimension is an attribute that describes a particular aspect of DQ and, if measured correctly, can show the total degree of quality of the data [24]. Due to different data environments, there are some differences in the definition of most dimensions. Metric is defined as a function that maps one or more data items to a numerical value that reflects the level of data quality [43]. Usually, when defining a dimension, the calculation method of the corresponding metric is also defined, and a dimension may correspond to one metric or multiple metrics. Metrics calculated quantitatively are objective, while metrics for qualitative assessment by data managers, users and experts can be subjective. Measuring dimensions with metrics mostly uses objective methods, but also some subjective methods, such as questionnaires in AIMQ, a combination of both subjective and objective metrics in DQA, or for statistical profiling in QAFD.

Wand et al. [92], Wang et al. [44], and Pipino [80] defined the six most important quality dimensions. Through the analysis, a basic suite of DQ dimensions can be defined, including accuracy, completeness, consistency, and timeliness, which are the focus of most authors [93]. Sidi et al. surveyed the studies on DQ management from 1985 to 2009 and summarized the definitions of 40 DQ dimensions [68]. Naumann et al. [94] defined holistic definitions of data based on a requirements investigation.

In the IoT context, Tilak et al. [95] proposed five metrics for the network layer of the IoT platform, including energy efficiency/system lifetime, latency, accuracy, fault tolerance, and scalability. Karkouch et al. [7] defined DQ dimensions for evaluating Wireless Sensor Networks (WSNs)nd RFID data, additional DQ dimensions for evaluating IoT data, and IoT domain-specific DQ dimensions, respectively, for a total of 11 dimensions. Teh et al. [5] indicated that DQ dimensions such as consistency and timeliness are not important in some IoT systems. Klein et al. [20] used five dimensions to evaluate the quality of sensor data streams, namely accuracy, confidence, completeness, data volume, and timeliness.

### 4.2. Classification of Dimensions

**Figure 4:**
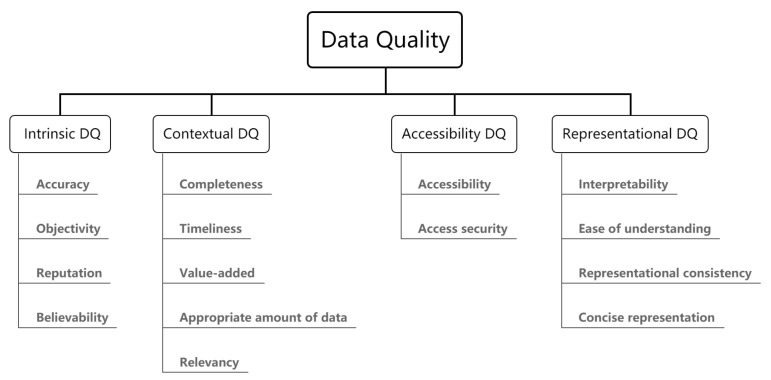
A DQ dimension hierarchy framework [44].

Wang et al. [44] initially listed 179 DQ attributes, and then proposed a two-level classification of DQ dimensions, where each of the four categories was further specialized into a number of dimensions. The four categories are: Intrinsic DQ, Contextual DQ, Representational DQ and Assesssibility DQ. The classification framework is shown in Figure 4.

The ISO/IEC 25012 [88] classified 15 DQ characteristics (characteristics are also referred to as dimensions or criteria) into two categories: Inherent DQ and System-Dependent DQ. Intrinsic characteristics focus on data domain values, data value relationships and metadata. For example, consistency and accuracy focus on these. System-dependent features focus more on the technical domain in which the data are used, such as precision of the device and portability. The 15 characteristics are:

- Inherent: accuracy, completeness, consistency, credibility, currentness.
- System dependent: availability, portability, recoverability.
- Both: accessibility, compliance, confidentiality, efficiency, precision, traceability, understandability.

### 4.3. Dimensions in IoT

There are a number of definitions for the quality dimension of data, and there are some surveys or reviews that summarize them. Only the definitions of DQ dimensions in the IoT environment or international standards are discussed below. There is no universal consensus on the exact implication of every dimension in the IoT environment. The various definitions provided in the IoT DQ studies are to be addressed below.

#### 4.3.1. Accuracy

Klein et al. [21] and Karkouch et al. [7] describe accuracy as the maximum systematic measurement error caused by static errors during the observation of streaming and static data as the numerical accuracy of the data values. The initial value of the accuracy is the absolute error of the sensor accuracy; the accuracy of the sensor can be obtained by consulting the specification provided by the manufacturer. For example, the DHT11 specification shows accuracy: ±1 ${}^{\circ }$C and ±1%, which means that the DHT11 temperature sensor has an absolute accuracy of 1 ${}^{\circ }$C, while the DHT11 humidity sensor has an absolute accuracy of 1%. This is only the inherent error of the sensor. Due to the specificity of IoT systems, environmental conditions, misplacement, calibration problems, and operational failures can also cause other errors that can lead to the degradation of data accuracy.

Geisler et al. [43] describe accuracy as a constant measurement error or an estimate of the quality of the measurement result, such as the confidence level of the result.

Perez-Castillo et al. [34] characterize accuracy as the level to which data have the property of correctly representing the true value of the expected properties of a concept or event in a given context. If the DHT11 humidity sensor reads 30% and the actual value is 50%, the accuracy of the humidity sensor may be low.

ISO 8000-2 [17] describes accuracy as a specification that controls the exactness of the approximate solution. Accuracy is divided into two categories: general accuracy that can be used for all measurements, and specific accuracy that can only be used for a specific measurements. ISO 25012 describes precision as two aspects: syntactic precision and semantic precision.

#### 4.3.2. Completeness

Geisler et al. describe completeness as the percentage of missing values or elements to the number of values/elements collected. As we can see from the definition, the metric is as the number of non-null values divided by the number of all values in the window (including NULL values) [43].

Klein et al. [21] and Karkouch et al. [7] considered completeness as the dimension used to describe the problem of missing values due to sensor faults or failures. A reference method was developed to calculate completeness based on the sampling rate γ [20].

Perez-Castillo et al. [34] considered completeness as the level to which the data have values of all intended properties and the associated entity instances in the specified use environment. The lower the completeness, the more data are lost from the device.

#### 4.3.3. Data Volume

The dimension of data volume is relatively well understood and easy to calculate, and is often used when assessing the quality of data streams. As shown in Table 4, we list two definitions and metrics of data volume. Perez-Castillo did not select data volume as an evaluation dimension in his study.

#### 4.3.4. Timeliness

Other time-related dimensions include currency and volatility. Currency focuses on how quickly the corresponding data are updated when they occur in the real world, and volatility indicates how often the data change over time [14]. The calculation of the metric can also be obtained from the definition of dimension. Unlike other DQ dimensions, timeliness can be calculated at runtime, but cannot be recorded, propagated, and processed during data processing. Table 5 shows three definitions of timeliness.

#### 4.3.5. Consistency

Geisler et al. [43] consider consistency to describe the level to which the values of an attribute adhere to the defined constraints. For example, whether the value is within a specific range, or a humidity sensor observes a negative value. Both rule evaluation and constraint checking can be used as consistency metrics.

Perez-Castillo et al. [34] argue that in a particular context of use, consistency primarily describes data whose properties are consistent with other data without contradiction. It can be a comparison of multiple data from a single device or a comparison of multiple devices that produce similar data. For example, two temperature sensors at the same location but two different temperature observations are obtained, which indicates a low consistency.

#### 4.3.6. Confidence

In an IoT environment, a single sensor placed somewhere may yield a low level of confidence, and the data are hard to confirm. If the confidence level of the data is low, it may be related to the inherent precision of the device or to the environment, which can be viewed in Section 2.3, Factors affecting IoT devices. Credibility is also used in some literature [34] as a replacement for confidence to describe this attribute of data quality. Several definitions of confidence are shown in Table 6.

#### 4.3.7. Other Dimensions

In addition to the above most common dimensions, some studies mention other dimensions for IoT DQ assessment, as shown in the Table 7. Most of the dimensions here are not commonly used, and the specific definitions are detailed in the relevant references; so here, we only select four of them for illustration.

Drop rate is a system performance metric that can be expressed in terms of the number of tuples reduced due to latency during stream processing. It can be used in stream data management systems that require the real-time processing of DQ.

Accessibility initially refers to whether the system supports people who need special support due to certain disabilities. In IoT environments, data accessibility is reduced or data from certain devices is inaccessible at a given moment due to network issues or user permission issues.

In IoT applications, some devices may provide inaccurate values due to their inherent low precision. For example, for weight sensors that only provide integer data, weight values with higher precision should provide at least three decimal places.

Low availability can result from insufficient resources to store sensor data. For example, to improve availability, sensor backups can be used, as well as backup sensors when one sensor has a problem, in order to ensure data availability.

### 4.4. Comparison of Dimensions

**Figure 5:**
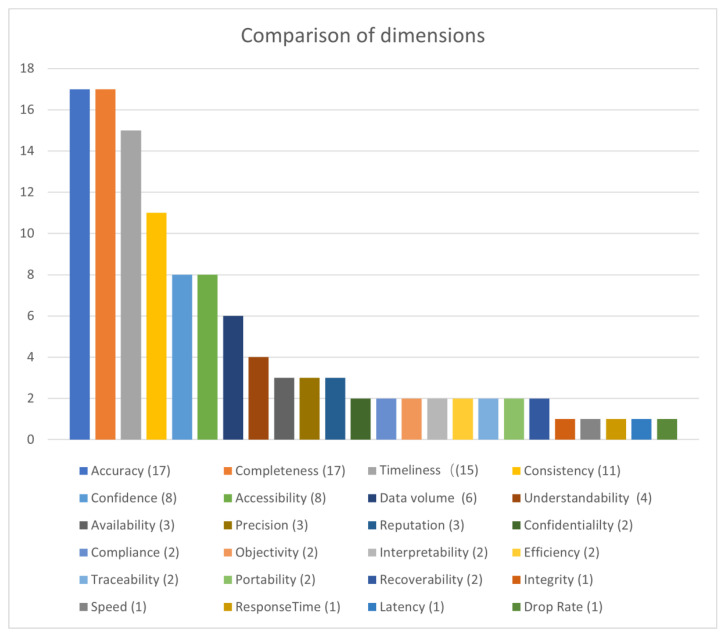
Comparison of dimensions.

Among the 21 papers, 5 standards were covered in the review in Section 3; a total of 19 papers and 2 standards entered the comparison, except for some methodologies that do not specify DQ dimensions, such as the MAMD Framework, MMPRO and DQIoT. We investigated the dimensions used in each study and counted the frequency of each dimension in the 21 studies, as shown in Figure 5. The number following the dimension represents its frequency of use in the survey.

Among these 21 studies, a total of 24 dimensions were involved. The most frequently used dimensions were accuracy and completeness, which were used 17 times. The dimensions that were used more than ten times include accuracy, completeness, timeliness, and consistency, which are the four dimensions that are most usedn DQ assessment whether in the general data domain or in the IoT environment. There are eight dimensions with a frequency of 2, all because Daqua-Mass draws on and customizes all the DQ functions defined in ISO/IEC 25012. Five dimensions were used only once.

### 4.5. Choice of Dimensions

Section 2.2 lists some of the possible issues in the IoT platform. This chapter discusses the correspondence between IoT DQ issues and quality dimensions. Unreliable data as a DQ problem type represent the inherent uncertainty of data items due to various factors. This uncertainty is related to the extent to which the values of the measured data items represent the actual values of measurement accuracy and precision. Both accuracy and confidence dimensions can be used to analyze these data items. Items with such DQ problems are described as having poor accuracy and confidence.

Low completeness and low data volume are the main symptoms of missing values. They are read as DQ problem classes as they are both converted to report the percentage of missing/missing values (e.g., NULL values) in the data stream.

Inadequate timeliness represents a special dimension of DQ, as they can be seen as an important symptom of DQ problems, i.e., degraded reads and unreliable reads. Indeed, on the one hand, outdated readings (i.e., failure to meet usage requirements in a timely manner) essentially mean that the readings requested by the application are not delivered in a timely manner.

Problems related to multiple sources of data usually manifest themselves as low consistency. In addition, the use of various data formats by data generation objects causes serious data representation problems, resulting in low interpretability and interoperability between incoming data streams.

The main influencing factors of IoT DQ and the correspondence of the six important dimensions are shown in Table 8. When evaluating DQ, appropriate dimensions should be selected based on the possible problems in the IoT system.

## 5. Conclusions and Future Work

In this survey, 21 methodologies or frameworks for DQ management containing DQ definition, assessment and improvement processes, and 5 international standards related to DQ were systematically investigated and compared. Unlike many other surveys, we first surveyed the definition of data types and IoT data types as a basis for subsequent comparison. Our review found that most most technologies specialized in structured and semi-structured data, while frameworks can rarely handle unstructured data. Most of the IoT data management frameworks focus on streaming data.

There have been many studies on generic DQ assessment dimensions; we mainly compared IoT DQ assessment dimensions, and finally found that completeness, accuracy, consistency and timeliness are the most important dimensions. Most of the frameworks used objective methods to calculate the metrics.

This paper additionally provides a guide for IoT DQ assessment that can help the reader identify the most appropriate methodology or framework. The options are made on the basis of many key factors, such as data type, data characteristics, possible affecting factors of the system, dimension and metric selection, etc., narrowing down the choice to the appropriate framework for a given situation. This review did not specifically summarize the improvement techniques in data management, and in the future, we hope to summarize the improvement techniques for IoT data as well.

IoT DQ management techniques are still young and many factors affect IoT DQ. Possible future research directions include customizing a more user-friendly DQ assessment methodology based on existing generic frameworks and employing some actions to improve DQ.

## Figures and Tables

**Table 1:** Classifications for data.

Ref.	Basis	Data Types	Description
[6,23,24]	Structure	Structured data	Data with formal schema definition; (e.g., relation tables)
[25,26]		Unstructured data	Generic sequence of symbols (e.g., video)
[27,28]		Semi-structured data	Data partly structured or have a descriptive without schema (e.g., XML file)
[24,29]	Change frequency	Stable data	Data impossible to change
		Long-term changing data	Data with very low frequency of change
[6,23,24,30]	Product	Frequently changing data	Dramatically changing data, (e.g., real-time traffic information)
		Raw data items	Data that have not been processed
		Information products	Results of manufacturing activities
[24,31]	Nature	Component data items	Semi-processed information
		Federated data	Data from different heterogeneous sources
		Web data	Data from the Web
		High-dimensional data	Big data
		Descriptive data	Consists of many tables with complex interrelationships.
		Longitudinal data	Time series data
		Streaming data	Data generated sequentially at a higher rate in a single source

**Table 2:** Layered distribution of factors threatening IoT DQ.

Layer	Affecting Factors	Examples	Error Types
Perception layer	Sensors Environment Security Privacy Network	Battery problems Precision limitation Mechanical failures Bad weather Device upgrades Unstable network Non-encrypted	Missing value [66] Incorrect value
Network layer	Network Environment Security Privacy	Unstable network Bad weather Security attacks	Missing value Incorrect value
Application layer	Streaming processing Security Privacy	Manually errors Obsolete schema definition Streaming operators	Wrong schema definition Misplaced value Broken join relationship Misplaced column values Missing record

**Table 3:** ISO standards related to data quality.

Standards	Components	Scope of Application
ISO/IEC 33000	Terminology related to process assessment; a framework for process quality assessment.	Information Technology Domain Systems
ISO/IEC 25000	A general DQ model; 15 data quality characteristics.	Structured data
ISO/IEC 15939	Activities of the measurement process; a suitable set of measures.	System and software engineering
ISO 9000	A quality management system; 7 quality management principles.	Quality management system
ISO 8000	Characteristics related to information and DQ; a framework for enhancing the quality of specific types of data methods for managing, measuring and refining information and DQ.	Partially available for all types of data, partially available for specified data types

**Table 4:** Definition and metric of data volume.

Ref.	Definition	Metrics
[43]	Number of tuple or observation values.	Amount of elements in the window.
[7]	The amount of raw data produced by the sensor with an initial value of 1.	The average data volume of data items contained in each DQ window.

**Table 5:** Definition of timeliness.

Ref.	Definition
[34]	Timeliness of data items relative to the application context.
[7]	The discrepancies among the timestamp generated by the data and the current timestamp.
[21]	The above two aspects.

**Table 6:** Definition of confidence.

Ref.	Definition
[21]	The extent to which the data are subject to random environmental interference.
[43]	Reliability of values or tuple.
[34]	The level to which users perceive the attributes of the data to be true and trustworthy in a given context of the use.

**Table 7:** Other dimensions.

Ref.	Dimensions
[43]	Drop Rate.
[34]	Accessibility, Compliance, Confidentiality, Efficiency, Precision, Traceability, Understandability, Availability, Portability, Recoverability.
[91]	Precision, Response Time, Latency.
[85]	Accessibility/Security, Clarity, Relevance, Cost/Benefit.
[7]	Ease of access, Access security, Interpretability, Duplicates, Availability, Duplicates.

**Table 8:** Quality issues related to dimensions.

Dimension	Quality Issues Related to Dimensions
Accuracy	Unreliable reading
Completeness	Dropped reading
Data volume	Data duplication; dropped reading
Timeliness	Multi-source data time alignment; unreliable reading; dropped reading
Consistency	Multi-source data inconsistencies
Confidence	Unreliable reading

## Abbreviations

The following abbreviations are used in this manuscript:

IoT	Internet of Things
DQ	Data Quality
ISO	International Organization for Standardization
PDCA	Plan-DoCheck-Act
SCP	Smart Connected Products
